# “The Good, the Bad and the Ugly” of Chitosans

**DOI:** 10.3390/md14050099

**Published:** 2016-05-17

**Authors:** Barbara Bellich, Ilenia D’Agostino, Sabrina Semeraro, Amelia Gamini, Attilio Cesàro

**Affiliations:** 1 Laboratory of Physical and Macromolecular Chemistry, Department of Chemical and Pharmaceutical Sciences, University of Trieste, Via Giorgieri 1, 34127 Trieste, Italy; bbellich@units.it (B.B.); gamini@units.it (A.G.); 2 Department of Life Sciences, University of Trieste, Via Giorgieri 1, 34127 Trieste, Italy; ilenia.d'agostino@phd.units.it (I.D.); ssemeraro@units.it (S.S.); 3 Elettra-Sincrotrone Trieste, Strada Statale 14 km 163.5, Area Science Park, 34149 Trieste, Italy

**Keywords:** chitosan, physico-chemical properties, molecular weight, degree of acetylation, conformation, antimicrobial activity, from gel to nanobeads, drug delivery

## Abstract

The objective of this paper is to emphasize the fact that while consistent interest has been paid to the industrial use of chitosan, minor attention has been devoted to spread the knowledge of a good characterization of its physico-chemical properties. Therefore, the paper attempts to critically comment on the conflicting experimental results, highlighting the facts, the myths and the controversies. The goal is to indicate how to take advantage of chitosan versatility, to learn how to manage its variability and show how to properly tackle some unexpected undesirable features. In the sections of the paper various issues that relate chitosan properties to some basic features and to advanced solutions and applications are presented. The introduction outlines some historical pioneering works, where the chemistry of chitosan was originally explored. Thereafter, particular reference is made to analytical purity, characterization and chain modifications. The macromolecular characterization is mostly related to molecular weight and to degree of acetylation, but also refers to the conformational and rheological properties and solution stability. Then, the antimicrobial activity of chitosan in relation with its solubility is reviewed. A section is dedicated to the formulation of chitosan biomaterials, from gel to nanobeads, exploring their innovative application as active carrier nanoparticles. Finally, the toxicity issue of chitosan as a polymer and as a constructed nanomaterial is briefly commented in the conclusions.

## 1. Introduction

**Figure 1:**
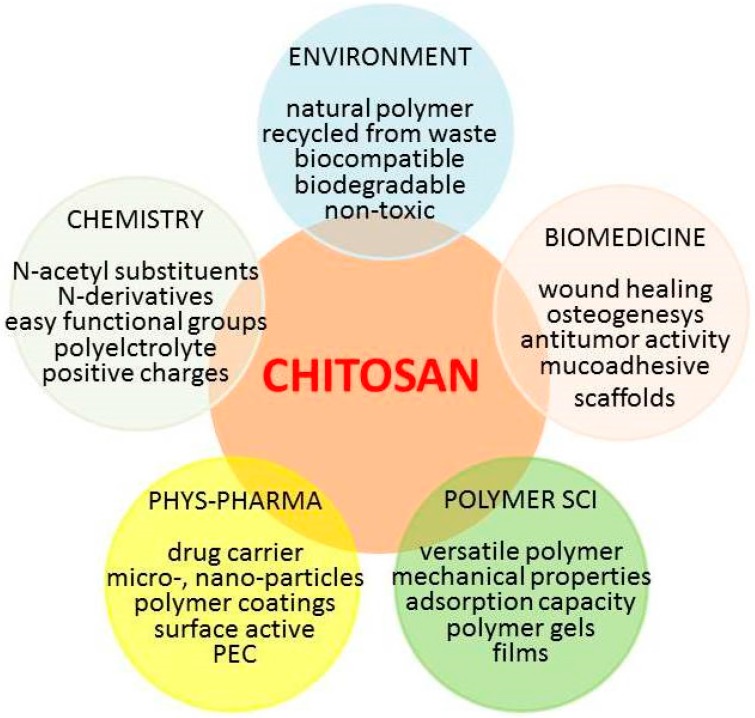
Schematic representation of relationship of chitosan properties with the pharmaceutical and biomedical applications.

Chitosan is one of the most commonly cited polymers in the scientific research dealing with a wide range of biopharmaceutical and biomedical applications including food science and technology. It has been strongly indicated as a suitable functional material in view of its excellent biocompatibility, biodegradability, non-toxicity, and adsorption properties. Originally known as a marine polysaccharide from shrimps and crabs, chitosan was primarily thought to be an easily accessible substance from the food industry waste. The original samples of chitosan were often still raw materials, with few exceptions of purification applied to chitosan in medical and cosmetic uses. The main areas in its characterization were in terms of quality (either purity or harmfulness), intrinsic macromolecular properties and physical form. Independently of the final human use, the first and most important issue was, and is still, related to the presence of impurities, metals and other inorganics, proteins, pyrogenic, endotoxic and cytotoxic agents, bioburden, all of which have received over the years erratic attention. The macromolecular characterization is mostly related to molecular weight and to degree of acetylation, but also refers to the rheological properties and solution stability (solubility, aggregation, and filterability). Last, but not less important in many applications, is the physical form of the powder or other morphology of the sample. Since nowadays there is a real boom of interest in chitosan, it is worth mentioning that all these characteristics have been amply and often repeatedly reported in literature in original research and review articles. Indeed, more than a thousand papers (1150) were published in the decade 1981–1990, increasing to more than five thousand papers (5700) in the decade 1991–2000, to about 23,100 in the first decade of this century. A current search of “Chitin and Chitosan” in research article titles gives more than 1600 results, almost one-half of which were published in the last 15 years; so why write another review? The simple answer is that another view-point can still be worthwhile, provided that some properties are cross linked and related to some critical specificity of chitosan in a novel way, as reported schematically in Figure 1.

Thus, having in mind to classify the “properties” of chitosan in categories as “the good, the bad and the ugly”, a scrutiny of its characteristics is here overviewed in order to introduce the theme of this paper. Among all natural polysaccharides the presence of the monomer glucosamine, either fully acetylated or partially deacetylated, is unique in the chitin/chitosan polymers. Whether explicitly highlighted or only mentioned as a secondary aspect, this structural characteristic is probably the basis of its outstanding biophysical properties and the key for a rationale of its applications.

In summary, the most simple and salient characteristics that make chitosan a beneficial and valuable polysaccharide are:

1. Chitosan is a linear natural polymer of glucosamine/acetylglucosamine that behaves as a polyelectrolyte with positive charge density at low pH.
2. Among the industrial polysaccharides, chitosan is an exception, being the only high molecular weight cationic polyelectrolyte, while polysaccharides are generally either neutral or anionic.
3. Chitosan is often claimed to be GRAS (Generally Recognized As Safe) and bioabsorbable.

The same versatile “good” characteristics are indeed also the reason for several undesired effects, starting from the most obvious one when dealing with copolymers, *i.e.*, the large variety of chemical composition in terms of comonomers. The large variety of chitosans differing in polymer size, degree of acetylation and possible chemical modifications increases exponentially. On the other hand, the simplicity of chemical modification is one of great strengths of chitosan. The “tunable” aspect of chitosan allows its optimization to give appropriate biomaterials for therapeutic applications, in principle enabling also the optimization of its biological profile.

The counterpart of the structural variability is in the difficult characterization of its (statistical) distribution of comonomers (glucosamine and acetyl-glucosamine) and in the unsolved question of how the compositional variability and the molecular weight distribution add complexity to the identification of beneficial properties of the final product (the “bad”). However, the studies of chitosan uptake, distribution and toxicity are indeed quite few, since most commonly in its drug delivery formulations, chitosan is the carrier or a functional excipient. Nonetheless, it is also evident that the inspection of chitosan interactions with some co-solutes or other biostructures may reveal some subtle effects both in classical solution thermodynamics as well as in biological applications, such as the “interactions with endotoxins”. All these undesired and often hidden effects are most appropriately classified as “ugly”, because they have frustrated scientists in their research efforts.

In the following sections the title issues are presented, relating and linking the chitosan properties, whenever possible, to some basic features and to advanced solutions for specific applications. Thus, after a first section in which tribute is paid to some pioneering work, the chemistry of chitosan is explored with reference, in particular, to purity, characterization issues and chain modifications. Then, the biocompatibility, antimicrobial activity and toxicity of chitosan in relation with its solution properties are reviewed. A section is dedicated to the formulation of chitosan biomaterials, from gel to nanobeads, exploring their innovative application as active carrier nanoparticles. Finally, the toxicity issue of chitosan as a polymer and as a constructed nanomaterial is briefly referred in the conclusions.

Particular attention is devoted to critical comments on the various conflicting experimental results, highlighting the facts, the myths and the controversies. The goal is to make the best use of the ***good versatility*** of chitosan, to learn as much as possible how to manage its ***bad variability*** and to tackle in the proper way its ***ugly undesirability***.

## 2. The Beginning

A short historical annotation may usefully illustrate the continuous efforts dedicated to increasing the knowledge of chitosans in terms of structure-properties relations and therefore to improving and developing their applications. Another pertinent preliminary comment is also necessary on the link, established from the very beginning, between academic research directly or indirectly related to commercial applications and the scientists involved in the R&D of companies present on the market in the fields of either generic chemicals or, more specifically, polymers. The scrutiny of papers dealing with chitosan properties gives rise to a permanent feeling that while its “potential” is continuously stressed, for many years this seemingly remained wishful thinking. Several factors concur with the idea that using chitosan as a market product could be beneficial. Some of these will be reported in the specific application discussed in this paper.

It is not strange that among the first studies on chitin and chitosan is the structural determination of the chain by diffractometry. The first X-ray diffactometric investigation of chitosan was carried out by Clark and Smith in 1936 [1] on fibers prepared from lobster tendon chitin by a solid state *N*-deacetylation. A subsequent X-ray pattern was obtained and fully analyzed [2] with a tendon chitosan prepared from a crab tendon chitin by similar deacetylation reported by Clark and Smith. Therefore, as mentioned by Ogawa in his review [3] “In spite of such early finding, the pattern was analyzed in 1997 [4], 60 years later.”

The relevance of crystallographic studies lies in the possibility that the chain conformation of chitosan can be affected by the chemical environment. Indeed, it has been shown that in the two polymorphs, hydrated (tendon) chitosan and anhydrous (annealed) chitosan, the crystalline structures consistently present antiparallel chains in extended two-fold helix, zigzag structure, with almost constant pitches of 1.034 and 1.043 nm, for the hydrate and anhydrous forms, respectively. This chain structure is similar to those found for chitin and cellulose. However, chitosan can easily change its solid state conformation from the extended two-fold to other structures by salt formation with acids. Although not comparable with other very flexible linear polysaccharides, such as amylose and pullulan, this moderate conformational flexibility is certainly due to the presence of free amino group on the monomer residue of chitosan; thus, the advantage of chitosan for its advanced tunable applications can be envisaged.

With regards to the more broad chemical and physico-chemical studies, very few papers were published on chitosan before 1970, some dealing with its characterization, a few on chemical reactions and derivative synthesis, and the largest number on its interaction with ionic species and the potential application in ion removal from waters. In this period, some research groups appear in the literature that dominated the later times. Between the 1970 and the 1980, some reactions (e.g., selective acylation) and properties of chitosan were assessed and many applicative properties of chitosan were explored. Among these works, it is particularly worth mentioning those on the hypocholesterolemic activity [5], the fungicidal effect [6], in addition to the reactions of chitosan to produce derivatives. A comprehensive review was offered by the First International Conference on Chitin/Chitosan held in 1977 in Boston [7], which signed the milestone for a continuous growing number of adepts in the chitin and chitosan forum. Thus, the great effort of the Muzzarelli group must be mentioned, starting with the characterization of metal chelating properties and the chromatographic uses of chitosan; as well as the contributions of the UK and Japan groups on the production and physicochemical characterization of chitosan gels.

In the following decade (1981–1990), an explosion of activities all around the world in the field of carbohydrate polymers gave rise to intensive studies in this area. In parallel, there was an increase in conferences dealing with industrial application of polysaccharides, with sessions in the very large meetings (e.g., International Carbohydrate Symposium, *etc.*) as well as some more specific thematic meetings (e.g., Grado 1981 [8] and Hoboken 1984 [9]). In the context of these meetings, several research groups emerged and the tie line for aggregation networks was marked.

In these and subsequent years, while continuous efforts were made on the preparation of well-defined samples (see, for example, [10]), many new ideas about the use of chitosan as biomaterial became explicit and other properties of chitosan and derivatives emerged in publications. The proposed uses of chitosan included the development of artificial skin, reconstruction of periodontal tissues, hemodialysis membranes, drug targeting and many other biomedical applications. The foreseen applications were always accompanied by sentences like “this novel biomolecule, biodegradable, and biocompatible, find applications in substituting or regenerating tissues” [11]. The similarity of chitosan structural characteristics with glycosamino-glycans, was interpreted as the reason for mimicking the functional behavior of the latter, while chitin was always structurally (or ancestrally?) associated with cellulose. In a slightly different direction, a derivative, *N*-carboxybutyl chitosan, was shown to display inhibitory, bactericidal, and candidacidal activities when tested against a few hundred cultures of various pathogens [12].

It is worth remembering that the first interest in the commercial applications of chitin grew in the 1930s and early 1940s, but took a backseat for many years because of competition from synthetic polymers. Large-scale production of chitin started in the mid-1970s when regulations were introduced to limit the dumping of untreated shellfish waste in coastal waters. Chitin was easily extracted from crab, lobster and prawn shells using solvents, and the production of chitin became an economical way to comply with the regulations and dispose of thousands of tons of shellfish waste. Although chitin has applications in creams and cosmetic powders and as a material for surgical stitches, the applications of chitosan expand to a multitude of areas: antibacterial lining for bandages and wound dressings, coating for seeds to boost disease resistance, agent to prevent spoilage in winemaking, in addition to its controversial use as dietary supplement (weight-loss by avoiding fat digestion). The specific area of food applications has been shortly reviewed in a 2016 paper [13], in particular for antioxidant and antimicrobial effects, which are useful in the food industry to improve food safety, quality, and shelf-life. It has been estimated that about the order of 10^10^ tons are produced annually by living organisms, although chitin and chitosan are recovered exclusively from marine sources. Applications of these polymers continue to grow and an estimated global market of chitin and chitosan derivative business was forecasted to exceed € 70 × 10^9^ by the year 2015 [14]. As reported in a news agency *“Not bad for bits of leftover lobster”* [15].

Before concluding this section, one point needs to be emphasized. The chain production of a ”food-waste-recycling-biopolymer” is compatible with achievement of ecological benefits and deserves further attention and effort. For chitin and chitosan, the environmentally-friendly materials produced from these renewable sources are sustainable and contribute to the creation of a circular economy, which is the current goal of an ambitious package put forward by the European Commission in December 2015 [16]. For the more specific aspects here concerned, the waste produced each year by the shellfish processing industries represents a practical challenge, since about 75% of the total weight of crustaceans (shrimps, crabs, prawns, lobsters, and krill) ends up as by-products. The current lack of acceptable waste management options creates a potentially large environmental hazard concern. All around the world, seafood wastes are thrown away at sea, burned, landfilled, or simply left out to spoil. Therefore, the extraction of chitin from shells and its use as it is or after further processing not only is a way to minimize the waste, but also to produce compounds with valuable biological properties and specialty applications, provided that all the raw material is correctly remodeled in well characterized chemicals. As recently pointed out in literature (“don’t waste seafood waste”) [17], the three major components of crustacean shells are calcium carbonate (20%–50%), proteins (20%–40%) and chitin (15%–40%), which could be separated by using an integrated bio-refinery with solvent-free mechano-chemical processes. Although the main objective of this work is focused on the biopolymer chitosan, it is mandatory to mention that within this sustainability view-point, several useful chemicals and primers can be obtained from chitin [18,19,20,21], giving further value to an atom efficient economy. Once again, a parallel can also be envisaged between strategies used, or to be implemented, in cellulose and chitin exploitation.

## 3. A critical Examination of the Physico-Chemical Properties of Chitosan Polymers

In order to emphasize, once again, the relevance of a correct understanding of the relation structure-properties in chitosan, let’s just mention in these first lines that chitosan composition has become a matter of great interest in patent claiming. Documentation for these claims lies on well-defined polymer chemical characterization and this concept will be further analyzed when discussing the difficulties encountered by regulatory agencies in approving chitosan uses.

The main outcome of a critical examination of the literature results is that often (although not always!) the correlation between properties of the nanoconstructs and the structure of the macromolecular components is missed. In particular, the impression is that the advantage offered by modulating the polymer structure is turned out in the disadvantage of having final products of remarkable variability. Therefore, aiming at exploitation of chitosan and chitosan nanoparticles in modern biomedical and pharmaceutical applications, some fundamentals of physicochemical properties of this biomacromolecule cannot be overlooked. The central “dogma” is that all useful applications can *in principle* be traced back to three molecular determinants: the degree of acetyl substitution, the molecular weight and its distribution, the nature and the fraction of substituents as pendant groups. In the more general applications, the additional step to be examined is the interplay of these molecular characteristics with the supramolecular interactions that provide size, shape and surface properties in the nanoconstructed patterns [22].

### 3.1. Chitosan as a Copolymer: Acetylation and Substitution

The first question concerns whether it is fully correct to speak about “chitosan” or better to use the term “chitosans”, making explicit the central concept that the term chitosans implies a series of copolymers that differ not only in the fraction of comonomers but also in the distributions and clength of the comonomer sequences. This aspect is well known in polymer sciences where the terms alternate-, block- and random copolymer have been introduced. It is also the case of alginate, a close polysaccharide, where the presence along the chain of the Guluronic (G) and Mannuronic (M) monomers with varying composition and sequence type, was amply recognized since long time [23], allowing to conclude, from ^13^CNMR studies, that the relative occurrence of G-centered triads deviated significantly from those predicted by first-order Markovian statistics [24]. It is also known that in chitosan the two monomers, *N*-glucosamine and *N*-acetyl-glucosamine, display quite different solution properties, given the ionic character in acidic conditions of the first monomer and the slightly hydrophobic terminal in the other. This intrinsic difference, getting amplified for block-type sequences of acetyl substitution, may have dramatic consequences on chain conformation and aggregation, a fact that has encouraged the research on more hydrophobic substituents able to self-assembling and collapse in micelle-type lipospheres. Furthermore, derivatization of chitosan by grafting side chains, like is done in quaternization or glycosylation of amino groups, gives rise to a ter-polymer (namely, constituted by acetylated, de-acetylated and substituted monomers), worsening, therefore, the already complicated situation.

Thus, the essential issue of chitosan composition is the proper sample characterization and, first of all, the correct determination of the degree of acetyl substituents, DA, (some authors use the term DDA, degree of de-acetylation). Over the years, several papers have referred on the determination of the degree of acetylation by physical and chemical methods, although the preferred method is often simple and easy to use, not the more accurate. The long list of methods include among all, titration [25,26], IR spectroscopy [27,28,29,30,31], UV spectroscopy [32,33], circular dichroism (CD) [34], NMR spectroscopy [35,36,37,38] and *N*-acetyl group hydrolysis [39]. As a preliminary and important annotation, the comparison of data obtained with different techniques often show discrepancies in the DA values. Indeed, the difference in solubility of samples having DA from 1 (chitin) to 0 (fully deacetylated chitosan) affects sensibly the results and no single technique could be adopted to cover the full range, pointing out that solution methods can be used for soluble chitosans, whereas 13C CP/MAS NMR [36,37] and infrared spectroscopy (as recently summarized in a review [40]) are used for chitin and highly acetylated chitosans. Still, each method presents advantages and difficulties either in the sample preparation and/or in running the measurements. As a novel technique, the calorimetric (DSC) determination of the distinguishable decomposition of amino and acetyl groups was recently published, providing results that are independent of composition and molecular weight [41]. Thus, DSC emerged as an accurate technique to determine the degree of N-acetylation in chitin/chitosan samples, the main advantages lying on the possibility of determining the DA in the whole 0 to 1 range, without solubilization, at lower cost in relation to NMR, and more accurately than with IR. However, all techniques, except for NMR based measurements, require an accurate weighing of chitosan. Therefore, moisture needs to be eliminated carefully and the purity of the samples must be determined separately. The degree of acetylation of chitosan determined by NMR is based on the fact that the O-Ac group is easily recognizable in the spectrum of chitosan recorded at room temperature and can be integrated and normalized by the integral either of anomeric protons or of other ring protons. More important is the possibility of generalization of the NMR method to study chitosan derivatives. An interesting application is that of glycosylated samples prepared with cellobiose, maltose and lactose residues. The glycosylation introduced a pendant made by an open sugar ring C1-linked to the amino group and C4-linked to the terminal glycosyl residue. The NMR spectra show small differences for all signals of the pendant groups except for the carbon 4 and the carbon 5, which reflect the configurational change from glucose to galactose moieties. The NMR on these samples provided a DA = 0.13 and a glycosyl substitution ratio ranging from 0.43 to 0.50 for the three derivatives [42].

**Figure 2:**
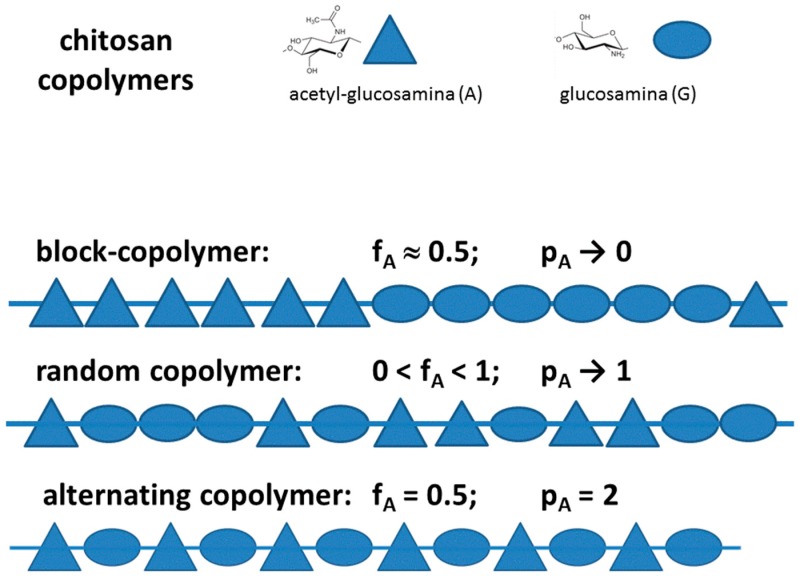
Schematic representation of three typical patterns of monomer distribution in a copolymer, here acetylglucosamine (**A**) and glucosamine (**B**). A perfect block-type copolymer with f_A_ = 0.5 should have a limiting p_A_ value = 0 for infinite length of blocks (top). A random distribution of the monomer (middle) is characterized a limiting p_A_ value = 0.5 for a value of f_A_ that can range around 0.5. The alternating copolymer, by definition, should have f_A_ = 0.5 and p_A_ = 2 (other values evidence structural irregularity). Thus, the scheme identifies limiting cases to interpret copolymer structure and underlines that nearest-neighbor effects give the three copolymer structures different physico-chemical properties.

Not less important for chitosan varying in pendant group substitution is the knowledge of the substitution pattern of the groups (including the acetyl groups) along the chain, as it was referred as a relevant property in copolymers. This is illustrated in Figure 2, where it is schematically evidenced why not only the fraction of comonomers (e.g., DA) but also their distributions and the length of the sequences of comonomers ultimately affect the polymer behavior, *i.e.*, each copolymer species is a “specific product”. The relevance of these characteristics increases with the difference in the chemical properties of the two monomer species.

In general, a statistical copolymer consists of macromolecules in which the sequential distribution of the monomeric units obeys known statistical laws, such as the Markovian statistics (the so-called random copolymer obey a zero-order Markovian statistics, coinciding with a Bernoulli distribution of the monomers). The only very non-random distribution of monomers is the one where monomers A and B alternate, Poly(A-alt-B), which can be clearly defined as a “new” polymer, different from Poly(A) and Poly(B) and which has rigorously fraction values f_A_ = f_B_ = 0.5. Another example of a copolymer distribution is that of “block copolymer” (PolyA-block-polyB), which is characterized by sequences of one monomer A interrupted by sequences of the other monomer B, the length of the sequences obeying to some *n*-order Markovian statistics (with *n* >> 1). All these different copolymer sequences can be initially identified by the monomer fraction, f_A_ = 1 − f_B_, and the probability of diads, f_AA_, f_BB_, f_AB_. A compact mode of providing the sequence probability has been proposed for the general case of copolymers by Bovey & Mirau [43] and recently used for chitosan [44] with the definition of the parameter p_A_ = f_AB_/(2f_AA_ + f_AB_) + f_AB_/(2f_BB_ + f_AB_).

The relevance of this quantification can be traced back to the original findings of Aiba [45], who studied two samples of chitosan with similar DA but different solubility properties. This author set up the hypothesis that the samples of chitosan with DA > 0.5 prepared from highly deacetylated chitin had to be considered as random-type copolymers of *N*-acetyl-glucosamine and glucosamine units, whereas the samples with similar DA but prepared from partial deacetylation of chitin had to be considered as block-type copolymers.

In conclusion, despite some different opinions in the literature and the generally assumed random statistics of residual acetyl substitution, the accurate determination of DA and of copolymer statistics is unavoidable. NMR determination of DA has been found to be precise and accurate also for the quantification of high DA, which is usually difficult to be measured with conventional techniques like IR or titration. Additionally DA can be calculated using different combinations of peaks in order to verify that the method is consistent. Some NMR techniques described in the literature are only limited by the solubility of chitosan, which depends on the DA and the molecular weight of the polymer. The relevance of DA on the solution properties of chitosan has been mentioned and, from a more general perspective, it should be stressed that all the literature results convincingly show that chain dimension and rigidity are related not only to the degree of polymerization, but also to the degree of acetylation (with a further obvious dependence upon pH and ionic strength). This point is discussed with reference to the molecular weight determination including the methods based on the knowledge of the hydrodynamic volume, having in mind the results reached on the cognate biopolymer, *i.e.*, hyaluronan [46,47].

### 3.2. Chitosan Copolymers: Molecular Weight Determination and Conformation

Before reporting on molecular weight (M) determination, another subtler question arises in these data for chitosans. Not only the problem of complete solubilization (1) or even the change in the macromolecular solvation (2) affect the M measurements, but also the correct interpretation of M can be vague if the degree of substitution is not known (3). Points (1) and (2) imply solution thermodynamic issues to be reviewed, since most of the experimental methods for M determinations deal with some solution properties measurements and a dependence of the solvent goodness through the Flory interaction parameter. Point (3) is of mere analytical origin and points out on the more classical definition of macromolecular chain length in terms of degree of polymerization (DP). As an example, a monodisperse chitin sample with DP = 1000, will display a molecular weight of 203.2 × 10^3^, while the same sample fully deacetylated, *i.e.*, the corresponding chitosan without *any* degradation, has a molecular weight of 161.2 × 10^3^, with a difference of about 20%. Therefore, even without degradation, a continuous change in the “measured” molecular weight is expected to occur upon deacetylation of chitin. This problem is much more impressive with large substituents as it occurs in the class of glycosylated chitosans [42,44]. The experimental determination of the M and M distribution of a series of samples of glycosylated chitosan with a constant DA = 0.13 and glycosyl substitution DS 0.42–0.50, is enlightening (Table 1).

Molecular weight and conformation analysis of these derivatives were investigated at UFT—Centre for Environmental Research and Sustainable Technology, Bremen, with a “triple detectors” size exclusion chromatography Viscotek system. The array, with a differential refractometer, a right angle (90°) (RALS) and a low angle (7°) light scattering detector and a four capillary, differential Wheatstone bridge viscometer, provided as final data the Molecular weight (M) and Molecular Weight Distribution (MWD) of fractionated samples, including viscosity of each fraction related to a given dilute polymer concentration [42]. The analysis of data allow to extract the weight average M_w_ and the parameters a and k of the Mark–Houwink–Sakurada (MHS) equation (*i.e.*, [η] = k M^a^). In particular, the most evident result is that, despite their overall similarity in the values and the trend of the viscosity, an increase (about doubling) of the molecular weight is observed in the glycosylated chitosans. This effect could be interpreted as a sign of chain dimerization, whereas simply arises from the derivatization reaction by which a pendant with M ≈ 320 is added on the chain about every two monomeric units, therefore doubling the original molecular weight in the absence of degradation. Therefore, these annotations claim for a re-examination of many published data of chitosan samples, in particular those with additional substitutions.

**Figure 3:**
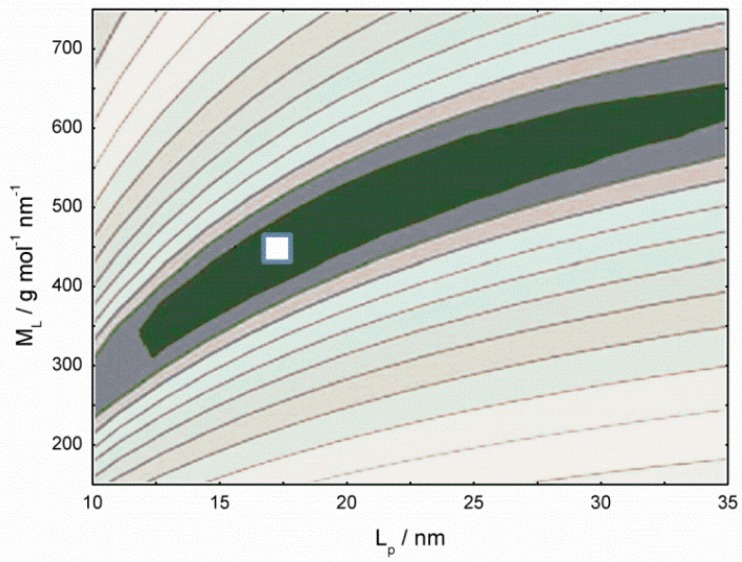
Descriptors of the chain conformation according to the Bushin–Bohdanecky equations. The solutions for chitosan are described in ref [48]. The diagram describes regions close to the target (dark color) and regions far from the target (pale color). The white square shows the coordinates of chitosan in the persistence length Lp (nm) *vs.* mass-per-unit length, M_L_ (g·mol^−1^·nm^−1^), plane (adapted and redrawn from ref [48]).

**Figure 4:**
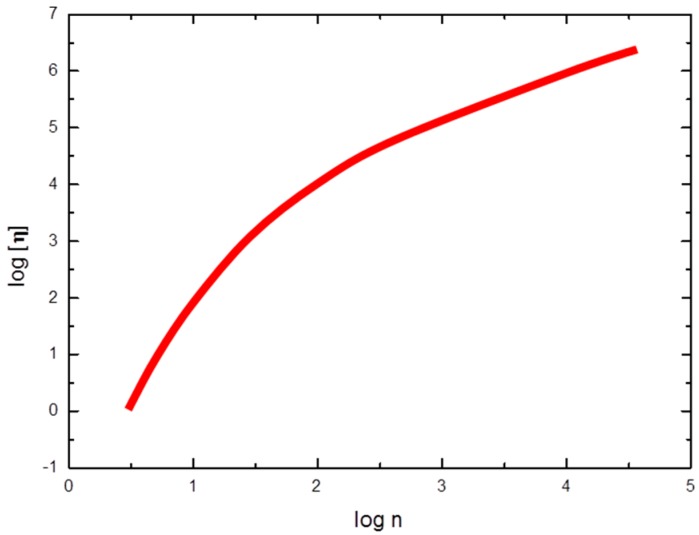
The dependence of intrinsic viscosity of chitosan and hyaluronan as a function of degree of polymerization (*n*) is reported as a master-plot (MHS log-log), normalized by the constant K (solvation constant). The emphasis is on the slope that increases with decreasing degree of polymerization n, *i.e.*, molecular weight M. The curve is drawn with the data of Figure 6b of ref. [47] for hyaluronan (0.5 < *n* < 4) and Figure 6.1 of ref [49] for chitosan (2 < *n* < 4.5).

As far as the conformation of chitosan with low DA is concerned, it is worth reporting that SEC data with triple detectors provide the full plot of log [η] as a function of M with the interesting result that the exponent coefficient *a* increases from 0.5 to 1 with the molecular weight decreasing from 5 × 10^5^ to 5 × 10^4^, in line with the literature independent results (Figure 3) about the concurrent estimation of persistence length and mass per unit length of chitosan (see Table 3 of ref. [48]) and with the findings for the moderately stiff hyaluronan chain. These results are collected in a sort of master curve and contain the fundamental dependence of the exponent *a* as a function of the degree of polymerization (Figure 4).

In conclusion, the correct polymer characterization and the robust interpretation of solution properties provide the basic knowledge for understanding the chain expansion behavior of the chitosans used for the preparation of nanoparticles, with the possibility of better tailoring the nanoparticle properties by selecting the suitable starting polymer.

Returning to the solution properties, points (1) and (2), in addition to direct methods for molecular weight determinations (e.g., those based on light scattering measurements), other indirect methods are largely used because they are simple and ready to use. Among these methods, the most popular one is the determination of the intrinsic viscosity, [η] which is log-log proportional to the molecular weight by mean of the Mark–Houwink–Sakurada (MHS) equation, [η] = k M^a^. The use of the equation implies, however, that a calibration is made on monodisperse fractions of the same polymer, under the same conditions of solvent and temperature. At this stage, it is clear that the calibration constants, k and a, depend on the very chemical structure of chitosan, starting from the value of DA and possibly on its distribution, since it has been recognized that the polymer solubility is affected. The solution to this problem was afforded in the past by changing the pH and the ionic composition. Empirical correlations have been reported to adapt the calibration constants of the MHS equation to a range of DA, pH and salt concentration. The use of the empirical relations, however, opens further questions about the compensation phenomena of chain expansion and solvation. Indeed, the analysis of the data of k produced by several authors, when plotted as a function of DA, shows the absence of a clear correlation that however could arise from some important difference in the acetylation patterns. The possibility that the samples may present a range of non-statistical sequence of acetylation patterns has been very recently presented [49,50,51].

### 3.3. Chitosan Solubility

On concluding this section on the intrinsic macromolecular properties of chitosan family, it is also necessary to add some words about the common practice of derivative preparation and the consequent changes in solubility that arise when substituents are inserted as pendant groups on the chain backbone. As a rule of thumb, polysaccharide solubility is affected by the chain linkage that governs the extension and the flexibility of the polymer. This is an important factor that makes the difference between polysaccharides otherwise undistinguishable on the basis of the rule “*similia similibus solvuntur*” (similar substances are miscible). This feature is clearly shown by comparing the configurational properties of several glucans, such as cellulose, amylose, pullulan, β-glucan [52,53], where flexibility and stability of the crystalline order play the major role. Therefore, for any given polysaccharide chain, solubility can increase (or decrease) depending on whether random (or regular) substitution is achieved and whether substituents can improve or not the interactions with the solvent. These concepts are schematically summarized in Table 2, where only a few examples are given to substantiate the above-mentioned rule of thumb. Chitosan is not an exception, as the changes in the degree of acetyl substitution and the introduction of other substituents (ionic, hydrophilic, and non-polar) conform to the above scheme.

Following these concepts, the issues of solubility of chitosan and chitosan derivatives can be focused in more detail and some issues of the past literature can be re-examined. Within the polysaccharide scientific community, the scarce water-solubility of chitosans, at neutral pH is a well-known and generally experienced fact. The solubility of chitosan(s) is an important concern representing a limit (if not an obstacle) not only to physical-chemical studies addressed to the structure-properties understandings, but also to its preparation and use as a polymer support or carrier material in biomedical applications, where neutral aqueous environment is often encountered.

It has already mentioned that the solubility of chitosan(s), *i.e.*, the entire family of partially deacetylated chitins with average degree of acetylation DA ≤ 50%, depends on molar mass, number and distribution of acetylation sites. These structural characteristics, in turn, are all related to the source of chitin and to the means of chitin extraction and deacetylation [54,55,56]. Besides the exploitation of other less common sources, the main industrial source of (α)-chitin is the shell (exoskeleton) of crabs or shrimps, while that of (β)-chitin is the squid pen. More recently, chitosan from chitin extracted from cultivated edible mushrooms, like *Agaricus bisporus*, has been made commercially available. This material, prepared by Kitozyme [57] and distributed by Sigma, is claimed to be a highly pure chitosan, ideal for wound healing and hemostasis, biosurgery and ophthalmology, scaffold and cell therapy, as well as drug delivery and vaccines. Since all commercial chitosans are far from an absolute purity, it is worth mentioning that a great difference in purity of chitosan may arise on whether the commercial product is used “as it is” directly in the human applications or it is subjected to physical and chemical modifications before end-uses. As a matter of fact, most procedures to modify chitosan by introducing pendant groups, or to process it by mixing with other polymers or chelating agents, imply “*de-facto*” manipulation that may introduce new contaminants or remove original contaminants. To the best of our knowledge, an analytical report on this particular aspect is lacking in the works published, while it will possibly be required in biomedical applications in addition to safety issues. Therefore, up-to-date information about accurate analytical reports is necessary and this specific point concerns both in-house purifications and commercial products. In particular, it would be desirable that data sheets of commercial products could be integrated with the macromolecular and characterization data discussed in this paper.

Indeed, keeping in mind that the amount of the residual *N*-acetyl glucosamine (GlcNAc) and the type of distribution, play a key role in determining the water solubility of chitosans [58,59], it is also necessary to analyze some solution molecular aspects. In particular, the role played by the glucosamine residues (GlcN) is generally referred to as improving solubility in acidic medium (*i.e.*, pH ≤ 6), due to protonation of amine group. On the other hand, the acetyl groups (and so the GlcNac residues) have been viewed as more hydrophobic entities that favor chain aggregation and negatively affect the water solubility [56,60,61,62,63]. However, while these concepts seem to be clear enough, the translation of the effects on a long chain is far to be naïve. Despite decades of the active research described, a clear relationship unambiguously linking the molar mass and the acetyl content and distribution to water solubility has been still elusive. Although a poorer solubility is encountered in homogenously re-acetylated samples with high DA content with respect to commercial ones [64], the homogeneity of DA distribution is reported to be one of the factors that increases chitosan solubility in aqueous medium as long as the solution pH is kept below 6. This can be reworded by saying that, even when the acetyl groups are randomly distributed, a pH lower than 6 is a prerequisite for chitosan solubilization [61,65,66]. On the contrary, the dissolution of chitosan samples with high acetylation content remains troublesome at a molecular level especially at high molar masses [64,67,68]. These findings were originally found by Anthonsen *et al.* [69] on fractionated chitosan samples with f_A_ = 0.01 and 0.60; by using several characterization methods, a bimodal molecular weight distribution was observed in which about 5% of the sample had a very high molecular weight. The presence of supramolecular structures revealed by electron microscopy and the possibility of partially reducing this aggregated fraction by ultracentrifugation and filtration were consistent with the positive virial coefficients obtained earlier from osmotic pressure measurements. Worth mentioning is that the presence of concentration dependent aggregates are reported also in chitosan of relatively low molar mass at low DA (12%) [70,71].

At this point, it is necessary a *flashback* with some general comments, since it is not a novelty that for a given concentration and solvent the increase of a polymer molecular weight adversely affects its solubility. More specifically, polysaccharides are known, as well, as gelling or thickening agents irrespective of their having charged or neutral backbones (e.g., agarose, amylose, carrageenans, xanthan, alginate). Some of their final applicative properties linked to biological function are also often exalted by the physical form of storage samples after chemical manipulation and purification and by the procedure of solubilization. In particular, for many polysaccharides, including chitosan, these physical forms and solubilization procedures refer to solution, powder, freeze-dried, air dried and presence or addition of simple salts, heating procedure, respectively.

Solubilization processes imply that a solid form of the material has been obtained and the material “history” is involved, making evident that precipitation, gelation or freeze-drying are not equivalent. Therefore, it is clear that the question about whether the acetyl moieties favor aggregation because they impart hydrophobic characteristics to the chain, remains unclear when not controversial, until both the short range and the long range effects are properly studied. For example, although it is suggested that acetyl groups affect the chain conformation by a continuous enhancing of chain stiffness upon increasing acetyl content [72], the interpretation of the previous literature data remains somehow conflicting. It is reported for instance and in contrast with previous observations, that DA strongly influence the stiffness of the chain as evidenced by the increase in MHS exponent a and radius of gyration Rg with DA, above a certain molar mass.

**Figure 5:**
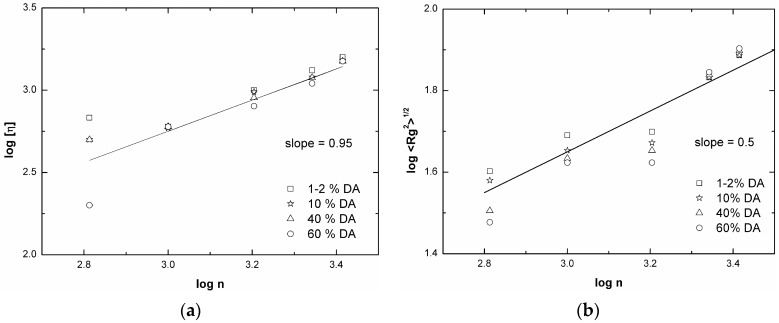
(**a**,**b**) Double logarithm plot of [η] and Rg values of chitosan samples with different DA values as a function of degree of polymerization, n. The data are recalculated from literature [63,64,72,73].

Still, an apparently consistent and reliable picture of the dimensional properties of chitosan in solution can be achieved by re-analyzing some original data of viscosity and radius of gyration and properly plotting these values as function of degree of polymerization, n, (Figure 5). The controversial deductions on chitosan behavior, might likely result from disregarding that the overall chain length characteristics play the main role on viscosity and light scattering experiments, from which conformation or shape are deduced. The generic Debye relationship for an isolated chain is between the viscosity and the number of residues ([η] ∝ n^1^) and a direct correspondence between M and n exists only for chemically identical residues, as already stressed above. Indeed, taking into account the value n, neither the parameter a nor the exponent ν (Rg ∝ n^ν^) seem to be much dependent on DA except for DA ~ 60%. Therefore, the fragmented picture that is given by the separated literature data [63,64,72,73] can be recomposed, as shown in the Figure 5.

As before, here the values of *n* = (M/Mru) are obtained from the experimental M and acetylation degree values [72,73] being Mru the average mass of repeating unit. The value of n can be identified as proportional to the chain contour length expressed in nm, being roughly 0.5 nm the average virtual bond length of the β(1,4)-d-glucose monomer (*i.e.*, the distance between two consecutive glycosidic oxygen atoms). On the other hand, the second virial coefficient is undeniably found to decrease with increasing DA and salt concentration, as expected for polyelectrolytes upon changing the effective charge density, being the effect the more evident the higher the M is [64,70].

Before concluding this section, a comment is still necessary of the mentioned changes of hydrophobicity as a function of DA. It should be clear that changes in local polarity by acetylation can scarcely affect chitosan properties as individual chains (very dilute solution). However, the influence of acetyl substitution can be more evident at high polymer concentration, where “hydrophobic” driven chitosan association can be observed. Under these circumstances, the presence of hydrophobic domains has been studied by mean of fluorescence using pyrene as hydrophobic probe, whose fluorescence spectrum is quite sensitive to the polarity of the microenvironment [71,74]. By ruling out the intrinsic acetyl contribution to the association tendency of chitosan chain, the authors assume that the formation of hydrophobic domains takes place at polymer concentrations close to and above the overlap concentration C*; for fully ionized samples with no added salt, this conclusion mainly indicates that the intermolecular character of the aggregation depends on the dimensional properties, *i.e.*, is independent from DA.

The scarce solubility of chitosan at neutral pH has been circumvented by modifying the polymer backbone with ionic or highly hydrophilic moieties. Chitosans bearing carboxylic, sulfate or *N*-alkyl groups have been synthesized for this purpose. Beside the enhanced solubility in aqueous medium, several additional features were disclosed by these modified chitosans. As a single mention, sulfate chitosan derivatives showed to be compatible with anionic polysaccharide like xanthan and the viscosity of the formed blends was found to be much greater than would have been expected from that of the individual components. Concerning the reductive alkylation route used to modify chitosan with the objective to reduce its intractability in neutral aqueous medium, the work of Yalpani and Hall [75,76,77,78] certainly deserves a specific mention; not only it showed the advantages of facility and versatility of the reaction procedure, but also it contained *in embryo* almost the entire “chitosan based biomaterials” research area whose development was still to come. It is not the scope of this review to discuss the synthetic approaches, since already reported in excellent comprehensive papers (for example, see ref [79] and references therein). However, the rationale of chitosan modification is that derivatization mainly aims at providing the following new performing properties. The first goal is to modulate physical-chemistry properties with the purpose to: (i) improve water solubility and blending ability with anionic biopolymers, enhancing and modulating rheological, hydration and compatibility properties of the new materials; and (ii) increase hydrophobic or emulsifying properties. The second goal is on the high-rated field of biological applications, with particular attention to derivatives that can impart specific biological activities to chitosan-based constructs through conjugation, and to those that can enhance antimicrobial activity. The intertwined manner with which these purposes can be found in the more recent literature might explain the continuous growth of active research on this subject. Driven by the desire of tailoring specific and complex properties, these activities led to a boost of studies on chitosan derivatives for potential applications in a very large variety of fields.

## 4. Biocompatibility, Antimicrobial Activity and Toxicity (Chemistry *vs.* Material)

Resuming the introductory section, since the very beginning of chitosan investigation, the properties of chitosan were interpreted in terms of a very promising technological material for a wide spectrum of applications [12,80,81,82,83]. In particular, besides all its recognized and foreseen properties, chitosan was claimed to possess the appealing feature of antimicrobial and bacteriostatic activity. Solutions, films and composites made of chitosan have been reported since the 1980s to be antimicrobial against a wide range of micro-organisms like bacteria either Gram-positive or Gram-negative, human oral and gingival pathogens [84,85] yeasts [86,87], algae [88,89] and fungi [90]. However, its effectiveness is continuously debated about whether it acts as a *bactericidal* agent, *i.e.*, capable to kill live bacteria or as a *bacteriostatic* agent, *i.e.*, able to arrest the bacterial growth without killing the microorganisms [91,92,93].

In a recent review, the current research in the areas of food, medical and textile industries was analyzed in order to summarize the progress in the study of antimicrobial properties of chitosan [93]. The comparison of the antimicrobial activity in relation to the structural differences in chitosan or chitosan derivative, the polymer physical form and the microorganism tested, made evident the difficulties of rationalizing the results. The conclusion to be shared is the variety of results reported by researchers even under apparently identical conditions, often due to the lack of standardization of the assay conditions. A further more general comment deals with the ambiguities in some of the studies concerning the assessment of chitosan antimicrobial potential, mainly deriving from inadequate data on the polymeric characteristics that affects this ability. Pointing at this ambiguity, in another recent analysis it has been reported that “Given the large number of proclaimed medicinal benefits of chitosan, it comes as no surprise that the literature is filled with conflicting reports about these medical potentials.” [94].

With this statement out of the way, it is obvious to recognize that the mechanism of chitosan antimicrobial activity has not been completely understood so far, since it depends on many intrinsic properties of the polymer itself and extrinsic factors related to microorganisms and environmental conditions. Among a long list of factors, it is worth reporting the molecular weight values [95], degree of acetylation (DA) [96,97], pH, type of derivative, solvent composition (medium) and, in particular, presence of metal cations [98], in addition to microorganism type and its outer surface charge [99,100,101], growth conditions, *etc.*

In general, it can be stated that the absence of reliable experimental determination of molecular parameters makes presently impossible to pinpoint the dependence of antimicrobial activity of chitosan on M or DA. As discussed in a previous section, the reliability does not reside in an intrinsic accuracy of the determination of the molecular parameters, but rather in the poor selection of the appropriate methods or even in the absence of any determination. In order to shed light on this issue, some literature findings can be tentatively organized about the mode of action, the killing mechanism and the interaction with cell components.

**Mode of action:** There are many hypotheses about how chitosan could exhibit its bactericidal activity, but almost all studies underline the determinant contribution of the poly-cationic nature of chitosan. Therefore, the electrostatic interaction emerges as a fundamental feature of the killing potential, since the interaction with the negatively charged microbial surface would dramatically affect the bacterial vitality. However, in view of the variability of the microbial surfaces, different organisms expose different more or less charged molecular patterns and respond to chitosan differently. This seems to be the case of gram-negative and gram-positive bacteria, although it is still uncertain which type is more sensitive. Some studies [12,102,103] indicate that the gram positive ones are more susceptible to chitosan than other micro-organisms, because of the presence of polyanionic teichoic acids on the outer surface [104,105]. Other studies reported that also gram negative are significantly affected by chitosan, implying a role of hydrophobic interactions by the exposed lipopolysaccharides [91].

**Killing mechanism:** Membrane structural perturbation by chitosan results from a series of studies by using a large number of different techniques, including the measurement of membrane polarity, evaluation of minimum inhibitory concentration, electron microscopy, transcriptome and proteome analysis. Based on the evidence of Raafat *et al.* it can be argued that the molecular mechanisms of action of chitosan in inhibiting or killing bacteria is definitely a complex process, which involves a series of interrelated events, that eventually lead to micro-organisms death [105]. First, the growth-inhibitory effect is shown in this study to be dose-dependent. A permeabilization of the cell membrane to small molecules and a significant membrane depolarization occur after treatments with the polymer, with consequent loss of cell integrity, stability and functionality. Moreover, a further implication on the cell components is disclosed by the changes observed in the expression profiles of the target organism (*Staphylococcus aureus* SG511 (*S. aureus*)), especially for those genes involved in regulation of stress, autolysis and energy metabolism [93,106].

**Interactions with the cell components:** Different opinions emerge in literature on the possibility that chitosan enters the cytoplasm. On one side chitosan is able to penetrate the cell and to interact with nucleic acid, thus interfering with the protein synthesis [107,108]. On the other side, the molecular mass of chitosan samples used in the experiments (not oligomers) has been questioned, making dubious its uptake. Alternatively, it has been suggested that high M chitosan deposition on cell surface could lead to a blockage of nutrients reducing microbial growth [98,109]. In a growth medium, chitosan might subtract micronutrients like essential metals (Ni, Zn, Co, Fe, Mg and Cu) to bacteria, inhibiting the production of toxin and important surviving molecules [93,110]. In conclusion, the proposed bacteriostatic activity of chitosan could arise from its well-known chelating ability.

## 5. Applications of Bacteriostatic Activity of Chitosan and Derivatives

Chitosan has been successfully used by researchers to exploit several applications in very diverse fields, in particular pharmaceutical (drug delivery, devices, and wound dressing), cosmetic, textile and food industries, as well as in agriculture and environment (waste-water purification). Some of the most interesting applications is based on the bactericidal potential of chitosan are summarized in the Table 3 of ref [93]. A summary of antimicrobial properties of chitosan and its derivatives is given, keeping in mind the general assertion that chitosan is considered as a GRAS (Generally Recognized As Safe) compound.

Multiple constructs/models of chitosan, and its derivatives, have been evaluated to express the bactericidal potential in medical field (e.g., post-surgery wound healing, patches and bandages as antimicrobial dressing after burning, antimicrobial coating of prosthesis) [111,112,113,114,115,116]. In the emerging field of nanobiopharmaceutics, antimicrobial chitosan nanoparticles have been studied for their enhancing effect of surface-to-size value. Thus, a vast number of works report the use of chitosan in form of nanoparticles, prepared in different conditions and blend, against various microorganisms [117,118,119,120,121].

In food industry, maintaining the quality and extending the shelf life of food products is mandatory. One of the most promising ways for effective preservation of food from alterations is using bioactive films as packaging. The use of active and/or edible bio-film based on chitosan alone, combined or enriched with different components such as plant extracts, other natural polysaccharides and antimicrobial peptides is emerging as an answer to such problems [122,123,124,125,126,127].

Finally, the use of antibacterial agents to prevent bacteria colonization is becoming a popular procedure in textile production, especially for goods employed in medical or hygienic services or sport-wears (odor-control textile) [128]. This antimicrobial supplementation is especially needed for natural fibers, which are more vulnerable to microbial attacks. Two examples, by Gupta and Haile [129] and Ye *et al.* [130], reported that a sensitive reduction of *S. aureus* (99%) was observed in modified chitosan embedded cotton.

## 6. Application of Chitosan as Delivery Systems

### 6.1. Chitosan Microparticles

The use of chitosan in pharmaceutical technology was originally conceived as an excipient for solid dosage forms, being used as coating, film-forming, mucoadhesive, disintegrant, tablet binder and viscosity-increasing agent [131]. The first investigations on the suitability of chitosan as drug carrier date back over twenty years [132]. Since the very beginning the use of chitosan was claimed to offer numerous advantages, such as high availability in nature, relatively low toxicity, and, above all, the presence of positively charged amino groups that enable both physical and chemical cross-linking [133]. In addition, after the seminal works of Yalpani and Hall [75,76,78,77], both the amino and hydroxyl groups have been exploited for a huge number of chemical modification [134].

Although among the several formulations the initial attention was focused on chitosan microspheres [135]. The spreading of nanotechnology shifted very fast the attention towards the nanoparticles, due to the numerous advantages offered by their size (in the nanometer range) [136,137,138]. Nowadays the choice of formulating micro- or nanoparticles is motivated by the type of performance required. For example, for intravenous delivery, only nanoparticles can be injected since microparticles would cause obstruction of blood vessels, while in case of pulmonary delivery, would microparticles have better efficacy, since nanoparticles would be exhaled [139].

**Figure 6:**
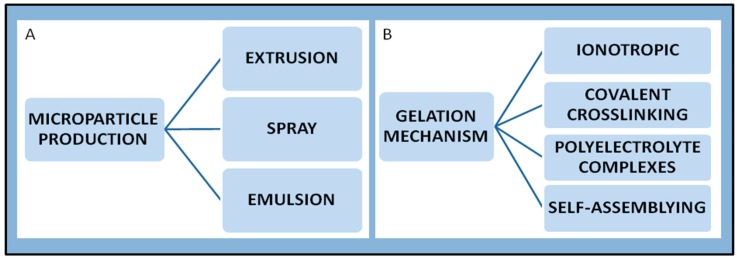
Graphical classification of the particles production (**A**) and mechanisms of gelation (**B**).

Micro- and nanoparticle production is the preliminary step for the physical generation of spherical domains, independently of the final stage of preparation. Thereafter, an appropriate gelation process is applied to stabilize the micro- or nano-domains. The most common procedures for micro-particle production are extrusion, emulsion and spray-technologies (Figure 6). The “extrusion” technique is widely used and, in the simplest case, can be performed by using a syringe with a needle. Several parameters, such as the diameter of the orifice, the flow rate, the viscosity of the solution, the distance between the hardening solution and the orifice, the polymer concentration and the temperature [140], control the size of the droplets and thus the final dimensions of the particles. The emulsion technique consists of dispersing a liquid in another one that is not miscible. Most importantly, it is possible to tune the size of the droplets by selecting the appropriate composition of the two phases, *i.e.*, the type and concentration of polymer and surfactant [141,142]. The spray-technologies are based on the atomization in heated gas (air or nitrogen) of a fluid material (solution, emulsion or suspension), which is followed by a fast removal of the solvent (usually water). The concentration of the polymer and the temperature of the system influence the final dimension.

### 6.2. Chitosan Nanoparticles

Over the years the interest in the formulation of chitosan nanoparticles dramatically increased, exploring new and innovative preparation methods [143,144,145]. Basically, the main mechanisms for chitosan nanoparticles preparation rely on a crosslinking process (either chemical or physical [146]), formation of polyelectrolyte complexes [147] and self-assembly of hydrophobically modified chitosan [145] (Figure 6). Other possible processes include reverse micelle, desolvation, precipitation/coacervation and emulsion-droplet coalescence [144,148].

**Figure 7:**
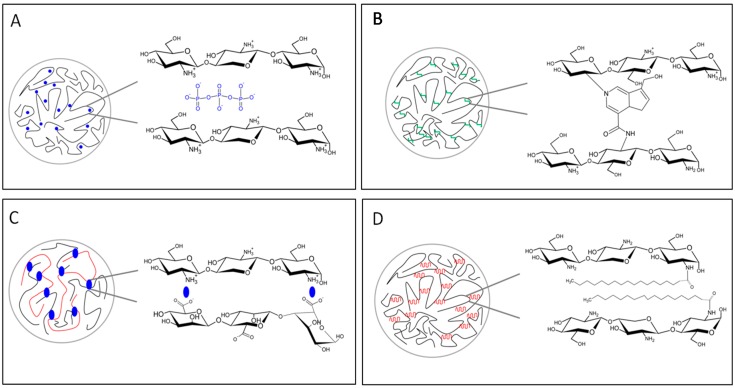
Gelation mechanisms for chitosan nanoparticles preparation: (**A**) ionic crosslinking; (**B**) covalent crosslinking; (**C**) polyelectrolyte complexation (PEC); and (**D**) self-assembly.

Chitosan crosslinking, such as a dropwise addition of a cross-linker to a chitosan solution, is considered an easy production technology to immobilize protein [149] or encapsulate different molecules [150,151]. Figure 7 summarizes the main characteristics of the gelation mechanisms. A classical covalent cross-linker is the glutaraldehyde; it acts as a bridge between two glucosamine units belonging to the same polymeric chain or to different chains. The amine group of the chitosan reacts with the aldehyde group of the linker leading to an imine bond via a Schiff reaction [147]. The concentration of glutaraldehyde strongly affects the textural properties of the final product [152]. The final structure results in an irreversible network with high mechanical properties, such as rigid network structures and high resistance to dissolution also in extreme pH conditions. One of the main drawback encountered is the high toxicity of the aldehydes [153]; the use of such systems is actually limited due to insufficient biocompatibility of the cross-linkers. Similar considerations can be done for epichlorohydrin [154]. A new crosslinking agent, genipin, which is found in gardenia fruit extract, is becoming an interesting alternative to glutaraldheyde [154,155,156,157,158,159,160,161,162].

Another possibility is the use of 1-Ethyl-3-(3-dimethylaminopropyl)carbodiimide (EDC), or similar compounds, which enable the formation of an amide bond, via an intermediate hydrazide derivative, between the amino group of chitosan and the carboxyl group of different macromolecules. EDC is also known as zero length cross-linker [163]. A valuable alternative is represented by carboxylic acids (especially from natural origin) [133,164]. All the linkers mentioned above can link chitosan with different polymers, such as gelatin [165], or other compounds, such as hydroxyapatite [166]). Attempts have been addressed to perform covalent modification using dextran sulfate [167]. Alginate too is potentially able to interact covalently with chitosan [168] producing resistant hydrogel. When choosing a covalent crosslinking method, it is important to check that the cross-linker does not bind also to the drug, to avoid formation of drug–polymer conjugates.

However, such covalent derivatives of chitosan are not considered as the best choice for drug delivery due to their lack of swelling [147] and absence of pH-dependence drug release. Chitosan is indeed appealing since it is a stimulus-responsive polymer [169].

The ionic crosslinking is also very appealing due to the presence of charged amino groups on chitosan in acidic conditions that allow to crosslink the polymer by using negatively charged ions, such as tripolyphosphate (TPP), which is the most widely used (note that this name entered incorrectly in the common language and is difficult to remove; chemically speaking it is a trimer and not a poly-phosphate, and even less a “tri-poly-something”). The interaction, commonly referred as ionotropic gelation, was firstly reported by Bodmeier [170] for the preparation of beads by dropping chitosan in a TPP solution and further extensively studied and described by Calvo *et al.* [171]. The ionic gelation takes place spontaneously via electrostatic interactions between chitosan chains and TPP [172]. This methodology of nano-particles preparation allows to encapsulate several types of biomolecules, from protein [173,174,175], to small drugs [176,177] and DNA fragments [178], and, because of its versatility, it was extensively studied and optimized [179,180].

Since the beginning, such mild conditions of gelation, appeared immediately very attractive for the encapsulation of labile drugs, such as proteins or peptides, characterized by poor bioavailability upon oral administration [181]. Within this frame, numerous studies have been conducted for the oral delivery of insulin [182], for the pulmonary delivery [183] and also for ocular delivery [184]. In addition to drug delivery, chitosan-TPP nanoparticles have been explored for many other uses. For example Du *et al.* explored the loading of various metal ions to obtain chitosan nanoparticles characterized by antibacterial activity [117]. This study evidenced that the antibacterial activity was significantly enhanced by the metal ions load.

Another very popular mechanism for the easy formation of nanoparticles is through polyelectrolyte complexations (PEC). Chitosan, the only natural polymer with a positive charge, is an attractive polymer for its ability to complex with a wide range of negatively charged polysaccharides by electrostatic interaction, forming PEC. More specifically, the interaction involves a phase separation where the solvent is excluded from the hydrophilic colloids. This can be achieved by adding a competing hydrophilic compound, such as a salt or an alcohol [185], and it is referred as simple coacervation, while complex coacervation occurs in a solution of two oppositely charged polyelectrolytes, giving rise to two immiscible liquid phases [186]. The phase rich in colloids is the coacervate, while the other phase is the equilibrium solution [187]. The interaction is likely to occur first via the long-range Coulomb forces, which provide for the primary strong binding energy, followed by the more directional short-range hydrogen bonds. The optimum condition for complex coacervation requires a constant control of pH. The mechanical properties and permeability of the products are strongly influenced by the properties of the starting material and conditions of reaction.

Chitosan is able to form PEC with both natural and synthetic polymers and since the early work published on PECs by Fuoss and Sadek (1949) [188], different studies and reviews have been published on this topic. PEC complexes obtained using natural biopolymers with opposite charge have been formulated for peptide [189,190] and protein [191] carriers but represent also an ideal colloidal carrier for DNA delivery [192]. Another emerging field of application is the delivery of antibiotics. Tobramycin loaded nanoparticles functionalized with dornase alfa demonstrated DNA degradation and improved nanoparticles penetration, thus increasing the efficacy of tobramycin [193].

In particular, among natural polymers, chitosan can interact ionically with several polyanion [194], such as alginate where the coacervation is due to interactions between the carboxyl functions of guluronic/mannuronic units of the alginate and the positively charged amine groups of the chitosan. A slight variation of this method is known as ionotropic pre-gelation reaction, as reported by Sarmento in a study for oral delivery of insulin [195,196]. This method, firstly reported by Rajaonarivony [197], involves an interaction among alginate, calcium and poly-lysine. The reaction consists in a first step characterized by an interaction between alginate and calcium ions, due to the gelling properties of alginate for the presence of guluronic residues [196], that are able to exchange sodium ions with divalent cations such as Ca^2+^, Sr^2+^ and Ba^2+^, forming the characteristic egg box structures. This step in the original study was followed by the crosslinking of pre-gelled alginate with poly-lysine, but in several subsequent works chitosan substituted poly-lysine [191,195,196,198,199,200]. Hyaluronan, another biocompatible polymer negatively charged that can be used to form complexes with chitosan [201,202] has the ability to bind several receptors as CD44 [203]. Hyaluronan can also be used as coating for chitosan-TPP complexation [180]. Further examples of complexation are represented by the reaction of chitosan with carrageenan [204], dextran sulfate [195,205], and xanthan [206]. Studies on similar interactions using negatively charged macromolecules, such as arabic gum [207], gelatin [208] and pectin [209] have been published.

A recent review [210] reports on the advances made in this field, focusing the attention on chitosan PEC’s with natural polysaccharides, such as alginate, hyaluronic acid, pectin, carrageenan, xanthan gum, gellan gum, arabic gum, and carboxymethyl cellulose, *etc.*, discussing also *in vitro* and *in vivo* data. On the other hand, synthetic polymers, such for example polyacrilic acid, have also been considered for polyeletrolytes complexation [211,212].

A sub-category of complex coacervation is represented by polyelectrolyte-colloids coacervate that has some advantage with respect to polyelectrolyte-polyelectrolyte coacervation in retaining the structure of colloids and in reducing the heterogeneity and configurational properties of the final system. In particular polymers-protein coacervates represent an interesting system for preservation of bio-functionality and are particularly important in enzyme immobilization, protein purification, antigen delivery and food stabilization [186]. Polyelectrolyte-protein coacervation [213] can occur between the amino groups of the chitosan and the reactive groups of heparin [214], BSA [186] and casein [215]. A change in the proportion between polyelectrolyte and protein can increment or suppress the coacervate.

Another appealing macromolecule is DNA, whose complexes with chitosan can be formed by simple mixing [216,217] and represent a promising non-viral vehicles for gene delivery [192,218]. A very recent review [219] reports on the promising use of chitosan as a non-viral nucleotide delivery system in spite of viral vectors that, although characterized by a high transfection efficiency, pose some safety concerns regarding immunogenicity and insertional mutagenesis. Thus, achievements towards this direction would be of great benefit for gene therapy.

Chitosan can be also modified with hydrophobic groups, resulting in grafted polymers that show a tendency to form inter- and intramolecular interactions in polar solvents by forming polymeric micelles that can be used to encapsulate hydrophobic drugs. The long polymeric chains interconnected with the hydrophobic molecules help stabilize the micelles protecting the internal drug. An abundant literature is available on this topic, investigating chitosan amphipatic behavior after the crosslinking with cholesterol [220,221], deoxycholic acid [222], stearic acid, and linoleic acid [223,224].

**Figure 8:**
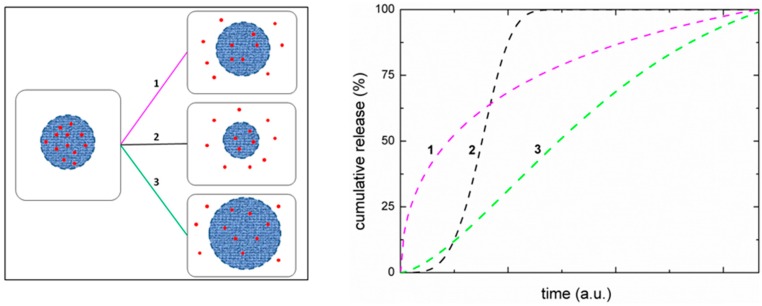
Example of release mechanisms (1: diffusion, 2: erosion and 3: swelling) and some related graphical trends according to mathematical models. Each mechanism is driven by the chemical structure of the network: elastic swelling and/or hydrolysis of cross-link, but also chitosan depolymerization by lysozyme action, in addition to the basic diffusion from the porous nanoconstruct.

Finally, it is worth to stress that a full knowledge of the physicochemical properties of the chitosan is required to obtain nanoparticles with tailored characteristics. The final structure of the network is the result of the contribution of different parameters, such as molecular weight and degree of acetylation, which affect the hydrophobic interactions and the network of hydrogen bonds. Xu and Du reported a direct correlation between chitosan MW and both encapsulation efficiency and release of BSA [173]. Similarly, formulation parameters, such as the ratio between chitosan and TPP [225] and operative parameters, such as stirring time and speed, affect the properties of the nanoparticles and their yield [226]. The release profile is a consequence of the behavior of the nanoparticles in the aqueous environment; more specifically the drug release occurs basically by three main mechanisms, diffusion, swelling and erosion (Figure 8), and it is strictly dependent on the type and degree of the crosslink. In case of a covalent cross-link, the permanent network obtained prompt for a drug release, which is mainly by diffusion, and the overall release profile will depend on the cross-linking degree. Similarly, the cross-linking density and the pH of the environment, as clearly reported by Berger *et al.* [146], influence also the swelling capacity. The mechanism of erosion is instead possible in presence of an ionic cross-linker. The kinetic of swelling and erosion will affect the initial part of the release curve determining a characteristic lag-phase. A huge amount of studies have been done in the field of model equations to describe the different several release profiles [227,228].

The improvements and modifications described so far will lead further progresses in pharmaceutical applications [229]. Many papers claim the potentiality of chitosan as carrier for drug delivery. Has this promise been fulfilled? Chitosan has been recognized as safe (GRAS) and approved for dietary use in Italy, Japan and Finland and only in 2008 a specific monograph was introduced in the European Pharmacopeia and in 2011 in the US National formulary. This of course has limited its use for the development of new drug formulations. Currently, chitosan is approved for wound dressing applications and cartilage repairing formulation. A recent review [230] on the use of chitosan as an absorption enhancer in nasal drug delivery formulations describes the ChiSys^TM^ technology for the delivery of peptides, proteins and small hydrophylic drugs. A chitosan-based formulation for nasal administration of morphine (Rylomine^TM^), at the moment in phase 2 (UK and EU) and phase 3 (USA) clinical trials, is expected to be released on the market in the near future.

The high level of knowledge on chitosan accumulated in the past years, both on the physico-chemical properties and on the use as drug delivery systems opens the possibility of many other applications [231].

## 7. Conclusive Remarks

The challenging point after reading so many articles and blending literature results with our own experience is recurrent in many lines of this article. It can be synthetized in two simple questions: How are ugly and bad tunable to good? How much we are willing to bet on a chitosan future? This final section attempts to resume the main issues that, to the best of our knowledge, should be addressed in the near future.

We would emphasize that the difficulties encountered in chitosan development as a marketed product in drug delivery and bioactive material could actually reside in the poor correlation between accurate chemical structure determination and its effective biological responses. An explicit reference can be made to references [42,43,44], where this issue has been recently reformulated. However, the main characteristics of chitosan to be considered, for example in the development of drug delivery systems, are not only the acetylation degree (DA), molecular weight (M), and purity, but also the relationships between internal and external parameters. Indeed, by stating that bioactivity and biocompatibility are both substrate- and host-dependent, it is clear that responses depend on investigated materials and on the experimental read-out used. In the case of chitosan, the understanding of material biocompatibility is hindered not only by the limited knowledge on the biological processes involved in material-cells interactions, but also by the poor assessment of the polymer characteristics. A recent review [222] addresses the general problem of the safety issue on chitosan uses, suggesting a more careful assessment of its safety in non-oral formulations.

A naïve answer is given by the fact that chitosan approval by the American Food and Drug Administration (FDA) is not a general definition as GRAS, but FDA and other regulatory agencies evaluate and approve materials with respect to specific applications. Not to forget that other relevant properties beyond the polymer itself are size, morphology, crystallinity, surface characteristics, degradation profile and additional products.

On the polymer side, the reported chitosan versatility and the variety of formulations add confusion to inexperienced researchers and regulatory scientists. For this reason, a thorough and systematic description of the chitosan used in a study should be either provided by the producer or be complemented by the research laboratories. Needless to say, the existence of expertise spread around the word and of large infrastructures should also be usefully utilized.

## Figures and Tables

**Table 1:** Physico-chemical properties of chitosan and chitosan derivatives (reported in [42]). Two commercial samples of Very-Low-MW and of Low-MW and three derivatives, a trimethyl-substituted (TM-chit), a galactosyl- (Gal-chit) and a glucosyl- (Glc-chit) substituted are reported. The molecular weight of the commercial LMW sample is given to be approximately 50,000–190,000 daltons based on viscosity. TM-chit contains also substitution on O6 and O3. The sugar substituent, gal or glc, is linked to ammine via an open sugar chain, since a disaccharide is the reactant. DS (non-acetyl substitution) determined by NMR. The column “Recovery” is a parameter relevant for the data to be representative of the sample investigated.

	DS	Mw	Mn	Mw/Mn	[η]	Rh	Rg	Recovery
		kg/mol	kg/mol		dL/g	nm	nm	%
VLMW-chit	-	30	18	1.7	0.7	6.4	-	94
LMW-chit	-	128	56	2.3	2.3	15.3	-	82
TM-chit	0.71	109	46	2.3	0.7	9.7	-	80
Gal-chit	0.50	473	102	5.1	1.9	21.3	50.8	99
Glc-chit	0.42	554	96	6.4	2.4	23.8	61.7	93

**Table 2:** Schematic role of structural and conformational features on solubility of polysaccharides.

Structural Features Change	Case-Polymers	Solution Behavior
chain linkage	cellulose	stiff and insoluble
	pullulan	flexible and soluble
side chains	curdlan	linear, insoluble
	scleroglucan	branched, soluble
non-sugar substituents	deacetylated gellan	gel with Ca, Mg
	native gellan (acetylated)	soluble with Ca, Mg
ionic groups (carboxyl)	Chitin	neutral, insoluble
pH > 6	hyaluronan	ionic, soluble, pH-depend.
